# Strategic Eye Movements are Used to Support Object Authentication

**DOI:** 10.1038/s41598-019-38824-z

**Published:** 2019-02-20

**Authors:** Jane E. Raymond, Scott P. Jones

**Affiliations:** 1 School of Psychology, University of Birmingham, Birmingham, UK; 2 College of Liberal Arts, Bath Spa University, Bath, UK

## Abstract

Authentication is an important cognitive process used to determine whether one’s initial identification of an object is corroborated by additional sensory information. Although authentication is critical for safe interaction with many objects, including food, websites, and valuable documents, the visual orienting strategies used to garner additional sensory data to support authentication remain poorly understood. When reliable visual cues to counterfeit cannot be anticipated, distributing fixations widely across an object’s surface might be useful. However, strategic fixation of specific object-defining attributes would be more efficient and should lead to better authentication performance. To investigate, we monitored eye movements during a repetitive banknote authentication task involving genuine and counterfeit banknotes. Although fixations were distributed widely across the note prior to authentication decisions, preference for hard-to mimic areas and avoidance of easily mimicked areas was evident. However, there was a strong tendency to initially fixate the banknote’s portrait, and only thereafter did eye movement control appear to be more strategic. Those who directed a greater proportion of fixations at hard-to-mimic areas and resisted more easily mimicked areas performed better on the authenticity task. The tendency to deploy strategic fixation improved with experience, suggesting that authentication benefits from precise visual orienting and refined categorisation criteria.

## Introduction

Authentication is a cognitive process used to detect close mimics of motivationally salient objects so that unnecessary or costly effort in approaching or avoiding them can be prevented. Indeed, judging the authenticity of many objects is a critical component of food (plant or prey) and predator identification for most animal species^1^. For example, some species use the evolutionary tactic of mimicking the appearance or action of more dangerous or more poisonous organisms to avoid predation^2^; yet, if the predator is able to detect that such prey is ‘counterfeit’, access to food sources that others may unnecessarily avoid can be gained^3^. For humans in the digital age where full-colour, high resolution graphic reproduction is ubiquitous, judging authenticity is important for approaching or avoiding stimuli used for socially and economically significant transactions or permissions. Specifically, authentication of complex, valuable documents (e.g., banknotes, passports), high value goods (including pharmaceuticals)^4^, and websites^5^ is an important cognitive operation that often implicitly and sometimes explicitly precedes object use. Yet, the cognitive processes that underpin visual authentication in humans remain poorly understood. To investigate, we examined visual authentication of banknotes, focusing specifically on the visual foraging strategies used for these highly familiar and frequently-handled stimuli. Although billions of people exchange banknotes every day, how these valuable objects are visually examined during authentication has not previously been reported.

Judging whether a banknote or other object is authentic is distinct from tasks involving rapid detection of a pre-defined target, as in widely-studied visual search tasks used in cognitive psychology laboratories. Typically, in those studies, the presence of a single visual target with pre-defined features must be detected in an array of other simultaneously presented objects or within a complex scene^6–8^. The categories defining target versus distractors (or background scene elements) are usually distinct and widely separated in at least one and often many dimensions. Eye movements during such tasks tend to be rapidly directed toward only those scene areas containing targets or target-like features^9,10^ showing that *a priori* knowledge of the search goal contributes to mechanisms controlling eye movements. In contrast, in an authentication task, the critical sensory information, or tell, used to confirm a counterfeit versus genuine decision is typically unspecified. A counterfeit tell can be the absence of an expected sensory detail, or the presence of an unexpected specific detail or feature. The tell or tells are necessarily imbedded within the object being authenticated. This means that the observer must judge object authenticity whilst processing a range of other object-related sensory signals that are highly consistent with internally generated neural predictions about the object derived from previous encounters with genuine exemplars^11^. As banknote counterfeit are rare in many communities, prior experience of how a counterfeit might differ in appearance from a genuine note is lacking for many banknote users. Moreover, counterfeits vary in their accuracy of mimicry, meaning that having identified a counterfeit tell previously might not facilitate detection of a subsequently encountered counterfeit. However, some people have some explicit knowledge of how to check a banknote (e.g., look for a watermark) and thus possess a prespecified notion of what to look for^12^.

Banknote authentication by the general public is also unlike well-studied medical diagnostic and baggage security screening tasks that involve extensive training of observers^13–17^. In both these highly-skilled tasks, rare visual targets that could belong to multiple, loosely defined categories must be discovered within complex scenes. Previous studies linking performance and eye movements in these tasks have shown that the acquisition of both explicit knowledge of targets and the accumulation of considerable visual experience with them leads to more efficient and target-directed eye movements, as well as better performance^15,17^. These studies not only support the notion that eye movements are instrumental for performance on these tasks, they also underscore the point that experience controls oculomotor orienting in context-specific ways.

In the case of banknote authentication, the general public have little or no experience with counterfeit and limited explicit knowledge of what the visual tell(s) identifying a note as counterfeit might look like^12^. In the absence of such information, how are eye movements controlled and can they reveal the state of knowledge held by users? We reasoned that, with no *a priori* knowledge, a useful eye movement strategy for authenticating banknotes might be to distribute fixations across the whole surface of the banknote without regard to content, searching for any discrepancy between the observed and a remembered genuine note. Such a search could be potentially exhaustive, defaulting to acceptance of the note as genuine only after a thorough search revealed no discrepancy. However, such a strategy would be reliant on a detailed and accurate memory of the genuine object. Considering previous findings of surprisingly poor explicit memory of details on everyday objects, like banknotes^18^, a widely distributed search strategy might be associated with poor authentication performance.

Alternatively, users could be more strategic in their visual foraging of banknotes if they were equipped with specific knowledge about the presence and location of specially-printed, harder to mimic elements, known as security features (e.g., holograms, colour changing patches, transparent or semi-transparent areas). The visual characteristics of banknote security features are intended to provide obvious authenticity tells to users whilst at the same time being particularly difficult to reproduce using commercially available technologies. If users had knowledge of a banknote’s security features, then they could prioritise orienting to these areas when authenticating a note, check for the presence of a specific visual feature as confirmation of genuineness, and thus produce better, more efficient counterfeit detection. Such directed fixations should be especially helpful considering that the critical authentication tell inherent in most security features is physically small and detailed, requiring precise fixation to be properly evaluated. However, it remains unknown whether security features are fixated more than other areas on banknotes because, to our knowledge, no previous study of eye movements during banknote authentication has been published. Nevertheless, security features that provide ostensibly obvious cues to authenticity are widely used as counterfeit deterrents for banknotes, passports, and other high value government-issued documents. Although they add considerably to document costs and thus burden the tax-payer, evidence that security features are used by the public has thus far been based solely on interview and questionnaire data^12^. Here, we use preferential fixation of security features as an objective index of users’ knowledge of security feature function.

Although it is well established that high-level cognitive mechanisms coding current goals and prior knowledge^19,20^ are able to control eye movements, there is also substantial evidence that gaze can be automatically captured by sensory configurations within a scene. For example, faces^21^, edges, curves, occlusions^22^, areas of sharp focus^23^ or those with perceptual salience^24^ are all know to attract fixations in a ‘bottom-up’ automatic fashion. If gaze during banknote authentication were captured in this way by a specific sensory feature, e.g., a portrait, that is easily and accurately mimicked, then propensity to fixate such a feature should be uncorrelated with authentication performance and be relatively resistant to effects of experience, e.g., practice or counterfeit encounters. In contrast, tendencies to strategically fixate useful, hard to mimic areas, such as a security feature, should be predictive of authentication performance and also become more pronounced with experience.

**Figure 1:**
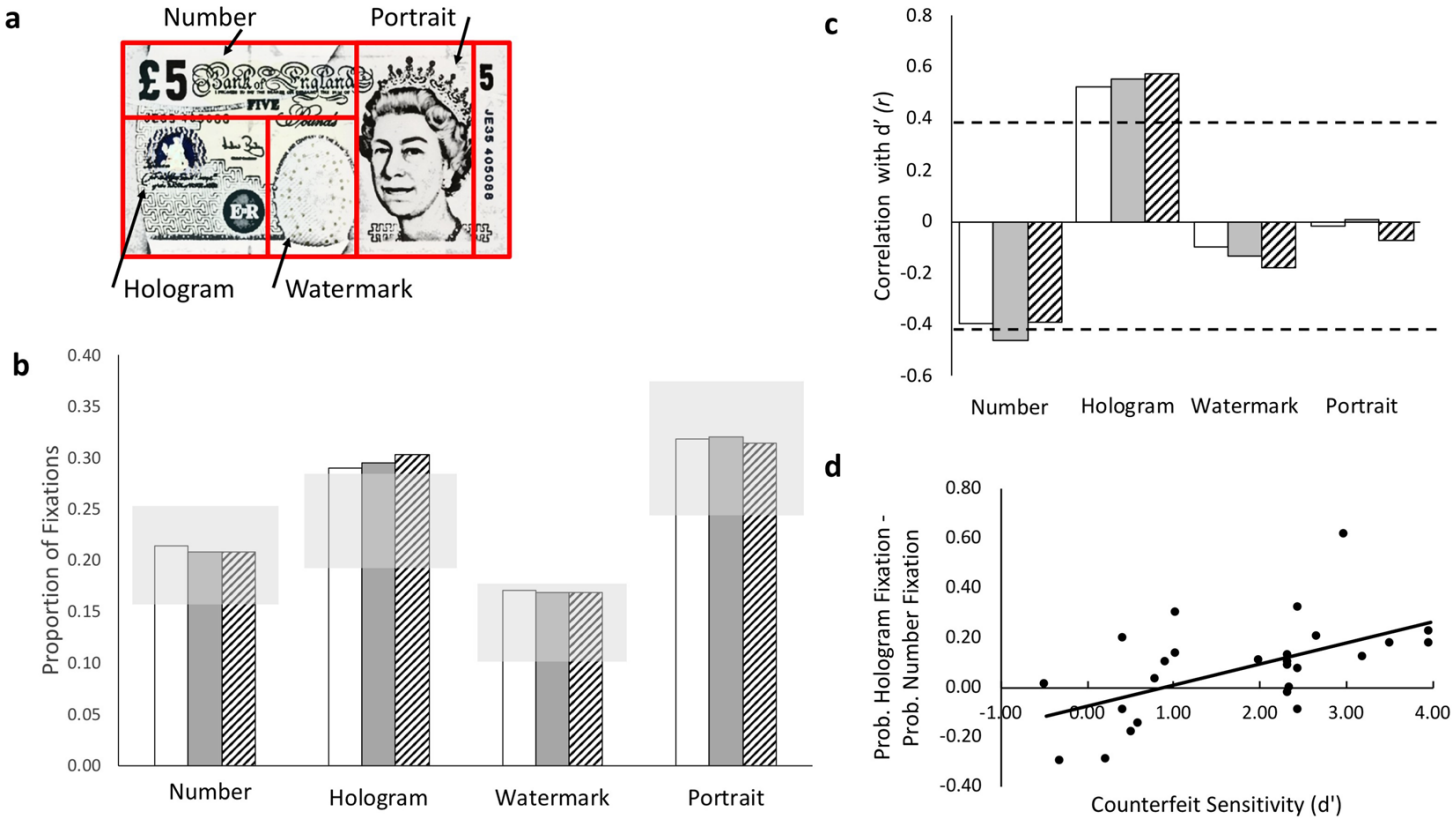
(**a**) An illustration of the £5 banknote used in the study. The solid lines (not visible to the participant) demarcate areas of interest (AOI) used to classify fixations. Each AOI occupied a different proportion of the notes’ total surface: Number, proportion = 0.21; Hologram, proportion = 0.24; Watermark, proportion = 0.15; and Portrait, proportion = 0.31. Only these four were used in the analysis; the remaining area on the extreme right edge was rarely fixated (<0.1% of fixations). (**b**) Group mean proportion of fixations directed at each AOI for genuine notes pre-counterfeit (white bars), for genuine notes post-counterfeit (grey bars) and for counterfeit notes (hatched bars). The light grey box indicates the upper and lower 95% confidence limits for values predicted if gaze distributions were determined by the AOI’s proportional area. (**c**) Pearson’s correlation coefficient (*r*) for the correlation between individual proportion of fixations and authentication performance (d’) for each AOI and note condition. Bar colours represent note conditions as in B. Dashed lines show the values needed for statistically significant positive (top line) or negative (bottom line) correlations (*p* = 0.05, 2-tailed, uncorrected for multiple tests). (**d**) Individual propensity to favour fixation of the Hologram AOI and avoid fixation of the Number AOI plotted as a function of counterfeit sensitivity (d’) score (where higher number mean greater sensitivity). *r* = 0.561, *p* = 0.003.

To investigate, we monitored eye movements of adults recruited from the general public as they authenticated as quickly as possible a series of genuine paper banknotes (not digitised images) interspersed with two counterfeit notes. Genuine notes used here were in general circulation at the time of the study; counterfeits were forensically recovered items; and both banknote types had similar signs of wear. To assess gaze prioritization in this task and how it was affected by experience, we assessed the distribution of fixations whilst participants authenticated successive presentations of genuine notes before and then again after encountering counterfeit. We compared the proportion of fixations directed at each of four different areas of interest (AOIs) on the banknote (see Fig. 1A) with the proportional physical area of those regions. If gaze were widely and indiscriminately distributed in search of counterfeit tells, then the proportion of fixations to each region should be statistically similar to that region’s physical proportional area^21,25^. However, if the proportion of fixations directed at a region were statistically greater or less than the region’s proportional area, then automatic capture or strategic control over orienting can be inferred. To distinguish the latter two possibilities, we examined effects of experience and correlated fixation probabilities with authentication performance. Such analyses were also used to assess how instrumental eye movements are for authentication. Preferential fixation of security features areas was used to index users’ knowledge of their function.

## Results

Twenty-six British adults judged the acceptability as quickly as possible of a series of 33 genuine and two counterfeit £5 UK banknotes, each presented upright against an opaque support (rendering the watermark invisible). The banknote’s primary visible security feature fell within the Hologram AOI and no other security features (aside from high resolution intaglio printing) appeared in any other AOI. Touching the note was not permitted. Unbeknownst to the participant the series was not random, but rather comprised a succession of 20 genuine notes, a single counterfeit, another 13 genuine notes, and, lastly, another counterfeit. This series created three note conditions: pre-counterfeit genuine, post-counterfeit genuine, and counterfeit (first and second) notes.

### Authentication performance

Both counterfeit notes were correctly rejected by 42.3% of participants; 46.1% rejected only one counterfeit; and 11.5% rejected neither, yielding an overall counterfeit rejection rate of 65.4%. The two counterfeits, although slightly different from one another, were rejected equally often (65.4% for both). Participants were 15.4% more likely to reject a counterfeit on the second versus first counterfeit encounter, suggesting an advantage of experience. On average, genuine notes were rejected on 14.5% of trials. The average individual *d*’ score (a conventionally used measure of counterfeit sensitivity, see *Methods*) was 1.79 (s.d. = 1.28), a value comparable to that reported in previous studies^26,27^. Neither cash handling experience nor sex had a significant effect of individual d’ scores (tested with independent samples t-test, 2-tailed; both *t*’s < 1, both *p*’s > 0.42). Age and d’ were non-significantly correlated (*r*(24) = −0.235, *p* = 0.258).

### Eye movements

Eye movements were recorded from the onset of each note’s presentation until an authentication decision was reported; data were analysed in two different ways. First, the distribution of fixations across the note during the entire viewing interval (without regard to fixation order) was analysed. This whole-interval analysis was used to indicate what information was acquired prior to decision-making. Second, the sequence of fixations (scan patterns) used when initially viewing the note was examined so that orienting prioritization could be investigated. For both sets of analyses, we compared eye movement behaviours before and after counterfeit exposure to assess effect of this experience. We ignored the individual’s response to the counterfeit banknote for these comparisons because neither a correct nor an incorrect response on this single trial is sufficient to infer awareness of the counterfeit’s tell(s). First, genuine notes were sometimes reported as counterfeit, meaning that correct detection of the counterfeit could be unrelated to awareness of its tell(s). Second, misclassifying the counterfeit cannot be used to infer lack of awareness of the counterfeit tell(s) because awareness could have emerged retrospectively upon viewing the next genuine note. To determine which eye movement behaviours were instrumental to authenticity performance we correlated each individual’s eye movement measures with his or her own d’ score, a criterion-free measure derived from responses on all trials.

For whole interval analysis, the proportion of fixations directed at each AOI was determined for each note condition and participant. Group means are shown in Fig. 1B. Fixation proportions were analysed by conducting repeated measures analyses of variance (ANOVA) separately for each AOI using note type as a within-subjects factor. Note type had no effect on fixation probability for any AOI (all F’s < 1 except for the Hologram AOI; *F*(2,50) = 1.20, *p* = 0.311, η_p_^2^ = 0.046). These findings suggest that neither experience (pre versus post counterfeit) nor small sensory differences between genuine and counterfeit contributed to choices over what information to check prior to submitting an authentication decision.

To test whether gaze was indiscriminately distributed across the whole note, one-way t-tests (2-tailed) compared group mean values for each AOI with the AOI’s proportional area. Only the Hologram AOI was fixated more often than expected by a distributed gaze strategy [*t*(25) = 2.41, *p* = 0.024], with all other AOIs attracting fixations at a rate predicted by their physical area. One-way t-tests (uncorrected for multiple comparison, alpha = 0.01) were also applied to individual data to determine how many participants showed this preferential fixation of the Hologram AOI. Indeed, only 46% of participants showed this propensity, suggesting that less than half had confidence that the banknote’s main security feature could provide a reliable authenticity tell. Interestingly, those that displayed this tendency were significantly younger (mean age = 34 years) than those that did not (mean age = 51 years), *t*(24) = 2.90, *p* = 0.007, 2-tailed).

To investigate whether an individual’s gaze distribution is predictive of authentication behaviour, each individual’s d’ score was correlated with their probability of fixating each AOI for each note condition. (See Fig. 1C). Tendency to fixate the Portrait or Watermark area was non-predictive of performance. However, propensity to fixate the Hologram AOI was found to be highly predictive of counterfeit sensitivity; propensity to avoid the Number AOI was also predictive, albeit less reliably. To determine if a combination of these latter two behaviours supported good authentication (even before a counterfeit had been encountered), the probability of fixating the Hologram AOI minus the probability of fixating the Number AOI was calculated for each participant for the pre-counterfeit genuine note condition. As can be seen in Fig. 1D, this difference was highly correlated with d’ (*r* = 0.561, *p* = 0.003, 2-tailed) and supports the contention that knowledge of the hologram security feature facilitated the acquisition of information useful for the task. Even higher correlations between these measures were obtained for the post-counterfeit (*r* = 0.608, *p* = 0.001) and counterfeit note conditions (*r* = 0.613, *p* = 0.001).

We also analysed the average duration (dwell) of fixations directed at each AOI during the whole viewing interval. When these data were analysed using a repeated measures analysis of variance using note condition and AOI as within subjects factors, we found that mean fixation duration depended on an interaction of AOI and note condition [*F*(6,72) = 2.72, *p* = 0.019, η_p_^2^ = 0.185, power = 0.832]. This effect resulted from note type determining the average dwell for the Hologram AOI. For the two genuine note conditions, average dwell in the Hologram AOI was nearly identical (Pre-counterfeit mean = 287 ms, s.e. = 8 ms; Post-counterfeit mean = 285 ms, s.e. = 10 ms), whereas for the counterfeit condition average dwell was 53 ms longer (mean = 339 ms, s.e. = 17 ms) than for either genuine note condition (both p’s < 0.006). No significant note condition effects were found for any other AOI. Longer mean dwell on the counterfeited versus genuine Hologram AOI is consistent with previous studies of eye movements during reading that showed prolonged dwell on words offering greater cognitive challenge (e.g., low versus high in word frequency or high versus low in syntactic complexity)^28^. Thus, the current observation of extended dwell to the counterfeit Hologram AOI suggests that the anomalous security feature found there initiated greater cognitive processing. Indeed, 62% of participants had longer than expected Hologram AOI dwell for counterfeit notes compared to their own mean dwell for genuine pre-counterfeit notes (i.e., greater than the 95% confidence interval, *p* < 0.05). Those showing this effect were not significantly different in age from those not showing this effect (*p* > 0.85). Interestingly, a positive correlation between dwell lengthening for this AOI and authenticity performance (d’) was found (Pearson *r* = 0.378, p = 0.034, 1-tailed).

On average, participants made 8.02 fixations per note (s.d. = 4.71). This value did not correlate significantly with *d*’ or age and did not depend on note condition (all *p*’s > 0.30). The average total accumulated time with steady fixation prior to decision was 2002 ms (s.d. = 1368 ms).

**Figure 2:**
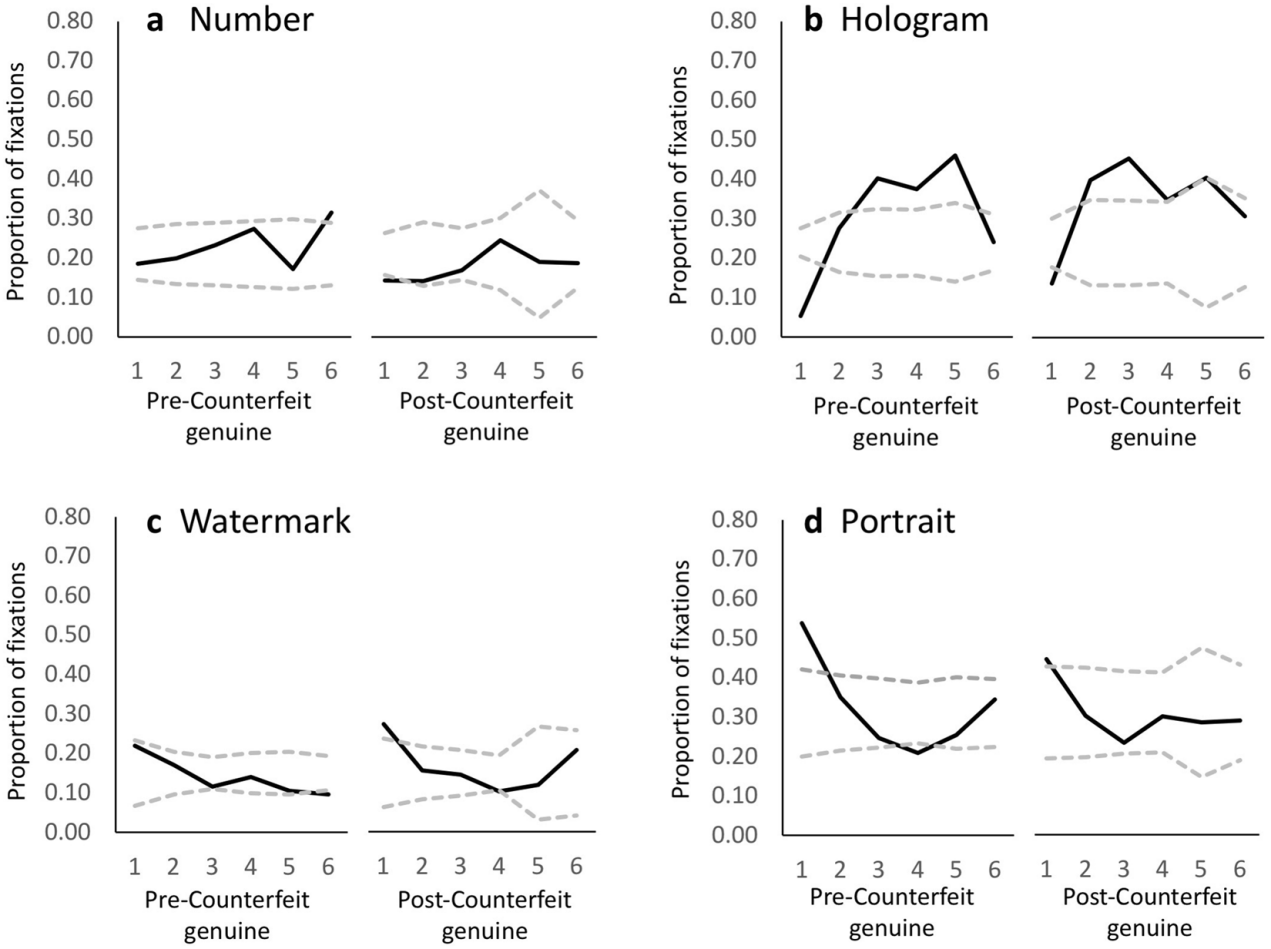
The group mean proportion of fixations allocated to each AOI. (**a**) Number; (**b**) Hologram; (**c**) Watermark; (**d**) Portrait) for genuine notes presented before and after a counterfeit encounter plotted as a function of fixation serial order (solid black line). Grey dashed lines represent upper and lower 95% confidence limits for deviation from the AOI’s proportional area; the distance between them reflects the variance in the data.

To investigate visual orienting prioritization, we next analysed scan patterns, i.e., the sequence of fixations. The proportion of fixations directed at each AOI for each of the first six fixations are plotted in Fig. 2 for genuine notes viewed before and after the first counterfeit exposure. Our statistical analysis, however, focused on the first four fixations only, as data for subsequent fixations was too sparse (<70% of trials yielding data). We conducted repeated measures ANOVAs separately for each AOI that used serial fixation order (1–4) and note condition (pre-counterfeit, post counterfeit) as within-subject factors. If gaze was indiscriminately distributed, then neither fixation serial order, note condition, nor their interaction should be evident and the proportion of fixations should be similar to the AOI’s proportional area. Contrary to this possibility, we observed significant effects of serial order and/or note condition for every AOI.

For the Portrait AOI, as fixation serial order progressed, fixation proportion markedly dwindled [*F*(3,72) = 14.75, *p* < 0.001, η_p_^2^ = 0.381, power = 1.0] from a group average high of 0.49 (s.d. = 0.27; greater than its proportional area, *t*(25) = 3.45, *p* = 0.002) for the first fixation to a low of 0.25 (s.d. = 0.19; not different from its proportional area, *p* = 0.15) by the fourth fixation. However, this serial order effect depended on Note condition [*F*(3,72) = 5.20, *p* = 0.003, η_p_^2^ = 0.178, power = 0.91] such that before counterfeit exposure, the tendency to initially fixate the Portrait AOI was 19% higher (mean = 0.538, s.d. = 0.28) than after counterfeit exposure [*t*(25) = 2.22, *p* = 0.036]. Nevertheless, this value remained high post-counterfeit (mean = 0.44, s.d. = 0.30; greater than its proportional area, *t*(25) = 2.31, *p* = 0.030). The probability of initially fixating the Portrait AOI before or after counterfeit exposure did not correlate significantly with d’, (before: *r* (24) = 0.09, *p* = 0.696; after: *r* (24) = 0.20, *p* = 0.333), nor did the size of the pre-versus post-counterfeit change in this probability correlate significantly with d’ (*r* (24) = −0.15, *p* = 0.461).

The proportion of fixations directed at the Watermark AOI also declined with serial order [*F*(3,72) = 7.30, *p* < 0.001, η_p_^2^ = 0.232, power = 0.98], starting from a high group average of 0.25 (s.d. = 0.20; greater than its proportional area, *t*(25) = 2.50, *p* = 0.019) on the first fixation and shrinking to a low of 0.12 (s.d. = 0.10; not different from its proportional area, *p* = 0.15) by the fourth fixation. However, this effect did not depend on note type [*F*(2.74, 65.77) = 1.788, *p* = 0.163, η_p_^2^ = 0.069, power = 0.42] and note type had no effect on its own (F < 1). The probability of initially fixating the Watermark AOI was marginally negatively correlated with d’ (*r*(24) = −0.371, *p* = 0.06), but thereafter correlations with performance were non-significant (p > 0.13).

For the Hologram AOI, the proportion of fixations significantly depended on serial order [*F*(2.26, 54.22) = 37.28, *p* < 0.001, η_p_^2^ = 0.608, power = 1.00], note type [*F*(1,24) = 4.47, *p* = 0.045, η_p_^2^ = 0.157, power = 0.53], and an interaction of these two factors [*F*(2.74, 65.74) = 3.43, *p* = 0.021, η_p_^2^ = 0.125, power = 0.75]. Specifically, before counterfeit exposure the Hologram AOI became the most likely AOI to be fixated by the third fixation (mean = 0.40, s.d. = 0.22; greater than its proportional area, *t*(25) = 3.79, *p* = 0.001), whereas after counterfeit exposure this occurred by the second fixation (0.40, s.d. = 0.28; greater than its proportional area, *t*(25) = 2.947, *p* = 0.007). Indeed, the Hologram AOI was 142% more likely to capture the second fixation after counterfeit exposure (*t*(25) = 2.34, *p* = 0.027) compared to before. Nevertheless, d’ did not correlate significantly with the probability of the Hologram AOI being the target of the second fixation before (*r* (24) = 0.19, *p* = 0.36) or after (*r* (24) = 0.29, *p* = 0.15) counterfeit exposure, nor did the size of the pre-versus post-counterfeit change in this probability correlate significantly with d’ (*r* (24) = 0.16, *p* = 0.44).

Unlike all the other AOIs, the proportion of fixations directed at the Number AOI did not depend on serial order. However, the proportion of fixations it attracted dropped significantly from a group average of 0.23 (s.d. = 0.14) before counterfeit exposure to 0.18 (s.d. = 0.13) after [*F*(1,24) = 5.58, *p* = 0.027, η_p_^2^ = 0.189, power = 0.62], suggesting an effect of experience. In neither case were these proportions different from the AOI’s proportional area (both p’s > 0.23). Interestingly, d’ correlated marginally with the probability of fixating the Number AOI before counterfeit exposure (*r* (24) = −0.37, *p* = 0.063) but was significantly correlated after (*r* (24) = −0.48, *p* = 0.014). The change in fixation proportion with note type was not predictive of d’ (*r*(24) = 0.05, *p* = 0.810.

Taken together these results not only argue against the idea that scanning banknotes is independent of content, they indicate that experience serves to modulate orienting prioritisation. Although the first and third fixations were most likely to be captured by the Portrait and Hologram AOIs, respectively, for both note conditions, the second fixation was more likely to be captured by the Portrait in the pre-counterfeit condition and by the Hologram in the post-counterfeit condition. This general pattern suggests that with experience, participants learned to suppress capture by the portrait, prioritise fixation of the Hologram area, and suppress fixation of the Number AOI. The latter area is probably habitually fixated in normal cash transactions where banknote denomination is important, whereas in the task used here, such information was irrelevant and fixation of this AOI unhelpful. Our data suggest that overcoming this fixational habit requires practice and/or active control over gaze.

As there were only two counterfeit notes tested in this study, data for analysis of sequential fixations of these notes were thin. Even so, we observed very similar scan patterns for counterfeits as for genuine notes. The Portrait AOI was the mostly likely location of the first fixation (mean = 0.50, s.d = 0.40) and the Hologram AOI was the most likely target for the second (mean = 0.40, s.d. = 0.35) third (mean = 0.52, s.d. = 0.44), and fourth (mean = 0.35, s.d. = 0.40) fixation.

## Discussion

The aim of this study was to determine how eye movements are used to visually forage for information when authenticating a complex object. To investigate, we examined both the distribution and sequence of fixations made during a repetitive, speeded banknote authentication task involving low denomination, genuine and forensically-recovered counterfeit banknotes. We sought to determine whether fixations were indiscriminately distributed across the banknote’s surface, intentionally directed at hard-to-counterfeit security features, and/or captured by sensory elements on the banknote. Additionally, we sought to determine whether control over eye movements is instrumental to object authentication. Collectively the findings from this study support the view that visual foraging during authentication is driven by a combination of strategic and automatic processes and that authentication performance depends on active, strategic control over fixation. Analyses of scan patterns show that as a group, gaze was initially captured by the portrait, then strategic control was weakly exerted to prioritize search of the hologram area, and this behaviour was followed up by a widely distributed check or search of other patterned areas of the banknote.

Only 65.4% of counterfeit presented here resulted in correct rejection in spite of participants having a lengthy interval to scrutinize each banknote. Eye movement analysis suggests that the reason why the critical authenticity tells on these notes was so often missed may result from people’s propensity to fixate areas of the note that are easily and accurately mimicked, at the expense of fixating the especially hard-to mimic hologram that was less visually compelling. Even though counterfeit and genuine notes appear maximally and obviously different in the Hologram area when genuine and counterfeit notes are viewed side by side, analyses of gaze distributions and scan patterns suggest that participants were generally unwilling to rely solely on the hologram’s appearance to make their judgement about the note’s authenticity. Although the hologram area was fixated more often than its physical size would predict, more than two-thirds of fixations were directed at other images and details on the note.

However, scan pattern analyses revealed that visual orienting was not random, at least initially. Participants showed a strong tendency to direct the first fixation toward the portrait and then over the course of the next two fixations a priority for the Hologram region become evident. Even so, the likelihood of fixating the Hologram at this stage was still less than 0.5. Other areas on the note, including the portrait, were consistently targets of the first four fixations, with the likelihood of fixation generally corresponding closely to the region’s proportional physical size. This pattern of response suggests that memory for the genuine hologram’s appearance was imprecise, or that participants lacked *a priori* knowledge or confidence that this feature of the banknote could effectively identify counterfeit. Nevertheless, the implication for this wide distribution of eye movement for the design of banknotes and other complex stimuli subject to counterfeit is that specially designed, hard-to-mimic security features should be as large as possible to maximize the likelihood of fixation.

Although visual foraging patterns demonstrated by the group as a whole are important for design of item such as banknotes, individual behaviour is more revealing of the cognitive processes that underlie visual authentication of objects. The data reported here show that individuals who exerted stronger strategic control over gaze by fixating the hologram with greater frequency and suppressed fixation of the Number AOI were more likely to perform better on the authentication task. This shows for the first time that eye movement control is instrumental to counterfeit detection and suggests that active effort in orienting is advantageous. It further suggests that perception of scene gist (or other global properties) that do not rely on precise fixational control are insufficient to support authentication, at least for complex objects such as banknotes, even though they may be critical components of object recognition and oculomotor guidance mechanisms^29^.

A clear relationship between eye movement control and authenticity behaviour was evident even before an actual counterfeit banknote had been encountered. This suggests that *a priori* knowledge that the hologram could provide a reliable tell may have been present in some individuals. Indeed, 61% of participants specifically favoured the Hologram AOI in either the second, third or both fixations (in the pre-counterfeit genuine note condition), suggesting they had specific knowledge that this security feature could provide a reliable authenticity tell. Furthermore, the probability of fixating the Hologram AOI in either the second and third fixation was significant correlated with d’ (*r* = 0.454, *p* = 0.020) and is suggestive of active strategic control. To the extent that propensity to fixate the Hologram AOI reflects knowledge of the security feature’s utility, these data suggest that educating the public about the presence and function of security features may indeed be beneficial.

Suppression of the tendency to fixate the Number AOI may also be viewed of evidence of active strategic control over visual foraging. Typically, when a banknote is encountered, fixation of the number is needed to verify the note’s denomination so that the transactions is completed appropriately. In the current task, denominating the note was not needed and possibly counter-productive. Indeed, tendency to fixate the number AOI was negatively correlated with authenticity performance and generally decreased as the testing session progressed. This supports the contention that the number AOI was unlikely to provide a good authenticity tell except via reduced print resolution in the counterfeit. Thus, suppression of a putative habitual response to fixate the number area should have required goal-directed control, a function probably mediated by the frontal cortex^30,31^.

Interestingly, tendency to fixate the portrait did not correlate with performance suggesting that information gained by scrutinizing the Queen’s face did not contribute to authenticity decisions, perhaps explaining why this tendency diminished slightly with experience. Nevertheless, the high probability of directing the first fixation to the portrait throughout the session is mirrored by previous reports of automatic visual orienting to a face image in other contexts^21,32^. Although gaze capture by the portrait was not unexpected, it has not previously been demonstrated in an authentication task where face processing is irrelevant nor with banknotes as scene stimuli. This observation has important implications for banknote design. Many of the world’s banknotes contain portraits for historical and cultural reasons, yet we show here that these images may degrade a banknotes’ counterfeit resilience by attracting gaze to a relatively easy to mimic banknote component. Banknote portraits are typically printed from finely detailed engravings using intaglio ink that yields a high contrast, high resolution image. The counterfeits used here did not use intaglio printing whereas the genuine notes did, giving the former notes lower image quality. Nevertheless, fixation within the Portrait AOI did not predict authentication performance, perhaps because rapid face recognition can be achieved even without high spatial frequency image details^33^. Such findings support other claims that perception of the portrait on a banknote contributes little to the perception of authenticity^18,34^.

In summary, eye movements used to garner corroborating evidence to support object authentication appear to reflect a combination of oculomotor control mechanisms. Automatic bottom-up mechanisms responsive to salient sensory features appears to drive eye movements initially, but by the second and third fixation, strategic control based on knowledge takes over when it is available. Participants who are especially successful at banknote authentication appear to direct gaze toward areas known to provide useful authentication cues and away from less informative areas. Others who are less successful at authentication appear to engage in a more random search of the entire note’s surface. This novel description of how visual orienting is used to support authentication not only has implication for banknote design, especially with regard to use of portraits and security features, and provides evidence that public education may be useful when novel security features are used on valuable documents or other objects, it also illuminates the importance of eye movement control when making precision judgements about object categories.

## Method

### Ethics statement

Participants provided written informed consent. The study was approved by the Science, Technology, Engineering and Mathematics Ethical Review Committee at the University of Birmingham and all research was performed in accordance with the relevant guidelines/regulations.

### Participants

Previous reports of gaze distribution to complex scenes tested between 13 and 30 participants per study. Using these figures, we recruited 31 adults from the community via online and poster advertisement to participate in exchange for £25. All were UK citizens, had English as a first language, and reported normal or corrected-to-normal vision and no history of neurological or mental illness. Four participants rejected more than 45% of genuine notes indicating their criteria for rejecting notes included signs of typical wear, even though they were instructed not to base decisions on this attribute. The remaining 26 participants (17 females) had a mean age of 43.3 years (s.d. = 15.3, range 23–65). Fifteen reported having had a job involving cash handling within the past five years.

### Stimuli

Thirty-three genuine £5, five genuine £10, and two counterfeit £5 banknotes were presented as test stimuli. Genuine notes were current legal tender at the time of the study issued by the Bank of England (Revised Series E; £5 first issued in 2002, £10 first issued in 2000) and were obtained from local cash machines and thus varied typically in soil and wear. The primary public-oriented (versus machine readable) security features on the front of this note were an image-changing silver-foil hologram (that alternated between two finely detailed images and reflected multi-coloured light), a watermark, and high-resolution intaglio printing in the portrait and calligraphy in the upper left. Counterfeit notes had been recovered forensically by the Bank of England, were judged as similarly “good” counterfeits by forensic experts, and showed a level of wear similar to that of the genuine notes. However, they had slightly lower resolution print and a very slightly different background colour. Both counterfeits included faithfully reproduced images (barring some loss in image resolution) of the signature, portrait, calligraphy, and patterned areas of the note and like the genuine notes each had a unique serial number. Both had a fake foil ‘hologram’ in the location of the genuine note’s hologram that had a less detailed central image that neither changed nor reflected coloured light. Both had small yellow micro dots on the watermark area (also present on the genuine note); one had a fake watermark; both lacked embossing and intaglio printing, and both were approximately 4–6 mm shorter on one dimension (one was shorter on the short dimension and the other shorter on the long dimension). The paper used to make the counterfeits was also lighter in weight. Only differences visible to the naked eye on the front of a statically viewed banknote served as potential cues to counterfeit in the present study.

### Apparatus

For the authentication task, each note was displayed using a custom-designed note holder. Notes were positioned at eye height, upright, portrait side facing the participant against an opaque card. They were illuminated from the front, preventing visibility of the watermark. An opaque screen with a 12 × 7 cm window positioned at the participant’s eye height allowed unobscured viewing of the note during each trial when the window was open and prevented view of the note, the experimenter’s hands, and the computer monitor (between trials). A chin rest and forehead restrainer were used to maintain a stable head position; each note was viewed from distance of 60 cm. The participant’s right eye was monitored remotely by an ASL Eye Link 1000 eye movement monitor that sampled eye position at 1000 Hz with a spatial accuracy of 0.25°–0.5°. A custom program constructed using E-Prime 2.0 software running on a Windows 7 PC computer was used to record responses, control timing intervals for eye monitoring, and generate calibration displays. Participants were tested individually in a quiet room.

### Procedure

For each trial in the authentication task, a single banknote was placed in the note holder and, after a ready signal, the viewing interval and eye movement recording interval was simultaneously initiated by the experimenter. The participant’s task was to report as quickly as possible whether the banknote was “good and should be retained in circulation” or “bad and should be withdrawn from circulation” by pressing the “1” or “2” key on the keyboard number pad, respectively. Participants were told: “Judge each note as ‘good’ or ‘bad’. A good note would be one that you would happily spend or accept in a shop. A bad note is one that you think should be removed from circulation”. “Bad” in this context to UK participants means counterfeit, excessively ripped, or stained beyond recognition. Participants were also told not to use typical signs of wear as a criterion for rejecting notes. Recording and viewing terminated with the participant’s response. Formal testing was preceded by seven practice trials using only genuine notes and a standard nine-point eye position calibration. The task comprised 35 successive note presentations in the following order: 20 genuine notes (pre-counterfeit phase), one counterfeit, 13 genuine notes (post counterfeit phase), and another counterfeit. Five eye-position recalibrations were interspersed during the series as was the presentation of up to five £10 genuine notes (to relieve boredom). Neither of these events immediately preceded or succeeded a counterfeit presentation. The presentation order of the two counterfeit notes was counterbalanced across participants; genuine note exemplars were shuffled randomly for each participant.

### Data Treatment

#### Good/Bad Judgments

The proportion of correct detection of bad (counterfeits) notes and the false alarm rate (judging a genuine note as “bad”) was calculated for each participant. As is conventional, a counterfeit sensitivity score, *d’*, was calculated by subtracting each individual’s z-transformed false alarm rate from their own z-transformed hit rate.

#### Eye Position Data

Eye blinks were removed using Eyelink’s blink removal function. Fixations were defined as a period of stable gaze lasting at least 100 ms between saccades; fixations less than this in duration were excluded. All data were excluded from trials involving a £10 note. These exclusions plus recording failures left an average of 16.0 genuine note trials (range of 10–20) for analysis in the pre-counterfeit phase and 10.6 such trials (range of 6–13) in the post-counterfeit phase. Data from one of the two counterfeit trials for one participant was also missing. The AOI for each fixation, its sequential order, and its duration (dwell) were recorded for each trial and participant (using a cut-off of 20 fixations, a value exceeded on 8% of trials). Then, averages and proportions were tallied separately for each participant and AOI.

### Statistical analyses

Greenhouse-Geisser corrections were applied when assumptions of sphericity were violated in all ANOVAs. One-way, 2-tailed t-tests were used to determine whether mean fixation proportion values significantly differed from those predicted by indiscriminate gaze control, i.e., from the AOI’s proportional area. Bonferroni corrections for multiple comparisons were applied unless otherwise noted. Pearson correlation coefficients (2-tailed, unless indicated) were used to test the significance of relationships between individual *d’* measures and eye movement indices. Alpha levels were set to 0.05 unless indicated.

## Data Availability

Data are available from UKData service ReShare https://reshare.ukdataservice.ac.uk/.
